# Exploratory associations between isometric strength, vertical jump performance, and psychomotor outcomes in elite traditional karate Kumite athletes

**DOI:** 10.3389/fspor.2026.1952574

**Published:** 2026-10-29

**Authors:** Patryk Niewczas-Czarny, Andrzej Kędra, Wojciech Wąsacz, Łukasz Rydzik

**Affiliations:** 1 Institute of Physical Culture Studies, College of Medical Sciences, University of Rzeszow, Rzeszów, Poland; 2 Department of Sport Science, Faculty of Social Sciences, Vincent Pol University, Lublin, Poland; 3 Institute of Sports Sciences, Faculty of Physical Education and Sport, University of Physical Culture, Kraków, Poland

**Keywords:** associations, combat sports, power, psychomotor abilities, strength

## Abstract

**Background:**

Traditional karate kumite requires the integration of physical, perceptual, and decision-making capacities. This exploratory study aimed to characterize selected neuromuscular and psychomotor variables and to examine their associations in elite traditional karate kumite athletes.

**Methods:**

Twenty-seven adult male members of the Polish national traditional karate team participated in this observational, cross-sectional study. Isometric strength of the elbow and knee flexors and extensors was assessed using the BTE PrimusRS dynamometer. Vertical-jump performance was assessed using the squat jump (SJ), countermovement jump without arm swing (CMJ_noAS), and countermovement jump (CMJ) with the Optojump Next system. Psychomotor performance was assessed using the TEST2DRIVE battery, including simple reaction time, choice reaction time, hand–eye coordination, and spatial anticipation. Associations were examined using Pearson correlation coefficients. Because 70 pairwise correlations were analyzed, the Benjamini–Hochberg false discovery rate (FDR) procedure was applied to control for multiple comparisons.

**Results:**

Several moderate associations were observed at the unadjusted level. The strongest association involving isometric strength was observed between right-arm extension strength and SPANT median motor time (r = 0.57; *p* = 0.002). Selected vertical-jump measures were also associated with psychomotor outcomes, including HECOR median motor time and CHORT response accuracy. However, none of the 70 correlations remained statistically significant after FDR correction (minimum adjusted q = 0.137).

**Conclusions:**

The observed relationships between neuromuscular and psychomotor variables should be considered exploratory and hypothesis-generating. The present findings do not provide sufficient evidence to conclude that vertical-jump performance is more strongly associated with psychomotor performance than isometric strength. Further studies using larger samples, body-mass-adjusted strength measures, and karate-specific upper-limb and whole-body reaction tasks are required.

## Introduction

Combat sports are highly popular worldwide. As competitive disciplines, they require athletes to possess advanced technical-tactical skills and above-average motor, physical, and sport-specific fitness to perform effectively in training and competition (1–3).

Traditional karate, particularly the kumite format, is one of the disciplines within this group. From a technical perspective, it involves a wide range of single strikes and combinations, including upper-limb punches and lower-limb kicks (1). Kumite is one of the most complex and demanding areas of sports training and competition in terms of integrating cognitive processes with the motor system. In the literature, sport kumite is classified as a classic open-skill discipline. It is performed in a highly unpredictable and dynamically changing environment in which athletes must continuously adapt their actions to the behavior and intentions of their opponents (2).

Unlike closed-skill sports, in which performance takes place under relatively stable conditions (e.g., weightlifting or gymnastics) and the emphasis is placed on reproducing a movement pattern, success in combat sports, including karate kumite, is determined by motor and technical-tactical preparation as well as by the perceptual and decision-making capabilities of the nervous system. From the perspective of sports theory (3), a key determinant of training and competitive success in striking combat sports is the ability to process visual stimuli rapidly and make accurate decisions under severe time pressure. The discipline is based on open skills and externally generated stimuli, and total response time comprises reaction time—the interval from detecting an external stimulus through information processing and movement planning—and the subsequent execution of the motor action (3). Systematic reviews indicate that elite martial artists do not always demonstrate a substantial advantage over novices in simple reaction-time tests. Their true functional advantage becomes apparent in complex reaction conditions that require information selection, anticipation, and the choice of an optimal defensive or offensive strategy (4). Sport-specific reaction-time and hand-eye coordination training has also been shown to directly influence the speed of scoring techniques in karate, such as the gyaku zuki punch (4). During actual tournament combat, the ability to maintain high vigilance and a short neural response time is also strongly affected by accumulating fatigue and the recovery strategies employed (5). However, even the fastest processing of information in the central nervous system will not result in scoring if the athlete lacks the motor capacity required to execute the technique immediately. From physiological and biomechanical perspectives, performing an effective attack or evasion within a fraction of a second imposes specific demands on the neuromuscular system (6).

To support effective occupational performance, institutions across various professional fields use advanced diagnostic methods. The TEST2DRIVE system has been applied in traffic psychology to assess drivers’ psychomotor and intellectual performance and cognitive processes. The system comprises 10 tests covering cognitive processes, personality, and psychomotor performance (7). The same psychomotor assessment method has also been used in sport, including among handcyclists (8), female handball players of different ages and competitive levels (9, 10), and kickboxers (6). During training and competition, athletes are continuously exposed to stimuli processed by the visual system (11). Owing to its comprehensive design, the system enables an overall assessment of indicators that may play a key role in athletic performance.

The traditional training paradigm in combat sports assumes that the development of maximal muscular strength is the foundation of physical preparation. This strength provides a basis for postural stabilization and energy transfer through the body's kinetic chain (12). Contemporary research nevertheless indicates that, unlike grappling combat sports such as judo, wrestling, and Brazilian jiu-jitsu, in which longer mesocycles focused on maximal strength may be justified because of close-contact combat and ground control, the ability to generate high force has a more limited transfer to striking dynamics in disciplines based on ballistic movements. Once an adequate strength base has been established, a different training stimulus should therefore be introduced (13). In this context, explosive power—a hybrid capacity resulting from the interaction of force and velocity—becomes particularly important. Rate of force development (RFD) is also a relevant indicator. A high RFD reflects the neuromuscular system's ability to maximally recruit and synchronize motor units during the first milliseconds (0–200 ms) of muscle contraction (14, 15). Explosive power, commonly assessed using standardized motor tests such as the squat jump (SJ) and countermovement jump (CMJ), is more closely aligned with the specific demands of kumite than maximal strength. This is because both ground-contact time during the initiation of an offensive action and the duration of the strike itself are too short for the muscles to generate maximal force (15). Previous research in male karate and jiu-jitsu practitioners identified body mass as the principal predictor of handgrip strength, whereas martial art practised and training experience differentiated reaction-time performance, suggesting that strength and reaction measures may have different underlying determinants (16).

Despite the rapid development of sport and exercise sciences, the literature contains relatively few detailed analyses of psychomotor performance, muscular fitness, and the relationships between these domains in karate athletes. Studies incorporating a broader range of variables describing this area remain scarce. In particular, relationships between strength capacities and reaction speed measured using advanced computerized tests, such as TEST2DRIVE, remain insufficiently understood. This issue is especially relevant in highly selected elite populations, such as national-team athletes. In these groups, the variance in somatic and motor outcomes is relatively limited because of rigorous selection, making it difficult to identify simple linear predictors of performance. To date, no comprehensive studies have examined the multicollinearity of power indicators such as SJ and CMJ and their actual relationships with individual components of psychomotor response in elite traditional karate athletes. Determining the extent to which neuromuscular potential is associated with choice reaction time and motor time has important practical implications for optimizing training aimed at achieving supercompensation before major championship events. Given the identified gap in scientific knowledge and the needs of coaching practice, such evidence may support the continued development of the discipline.

Importantly, the neuromuscular and psychomotor measures used in the present study represent different motor domains. Vertical-jump tests primarily assess lower-limb performance, whereas the TEST2DRIVE tasks require responses performed with the index finger of the dominant hand. Therefore, the rationale for examining these variables was not based on an assumption of direct biomechanical transfer between lower-limb power and finger-movement speed. Rather, the study explored whether general neuromuscular characteristics co-varied with psychomotor performance in a highly trained athletic population. Measures of upper-limb power, punch velocity, or whole-body reaction performance would provide greater task specificity and ecological validity but were not included in the present study.

Accordingly, this exploratory study aimed to characterize selected psychomotor and neuromuscular variables and to examine the associations between isometric strength, vertical-jump performance, and psychomotor outcomes in members of the Polish national traditional karate team competing in kumite. Given the differences between the motor effectors and tasks assessed, no direct biomechanical transfer between vertical-jump performance and finger-movement speed was assumed.

## Methods

### Ethics approval and informed consent

The study was approved by the Bioethics Committee of the University of Rzeszów (approval no. 58/05/2026; approved on May 29, 2026). In accordance with the Declaration of Helsinki, all participants were informed about the study objectives and procedures, potential risks and adverse events, and their right to withdraw from the study at any time without providing a reason. Before participation, all athletes provided written informed consent.

### Study design

This observational, cross-sectional, correlational study was designed to determine the relationships among muscular strength, lower-limb explosive power, and psychomotor reaction parameters in elite traditional karate athletes. Muscular strength was assessed using isometric force parameters recorded with a PRIMUS dynamometer. Lower-limb explosive power was evaluated using the squat jump (SJ), countermovement jump (CMJ), and countermovement jump without arm swing (CMJ_noAS). Psychomotor reaction parameters were assessed using the TEST2DRIVE system.

Because the study was cross-sectional and correlational and focused on associations among the assessed variables within a homogeneous group of elite athletes, no external control group was included. The study procedure is presented in Figure 1.

**Figure 1:**
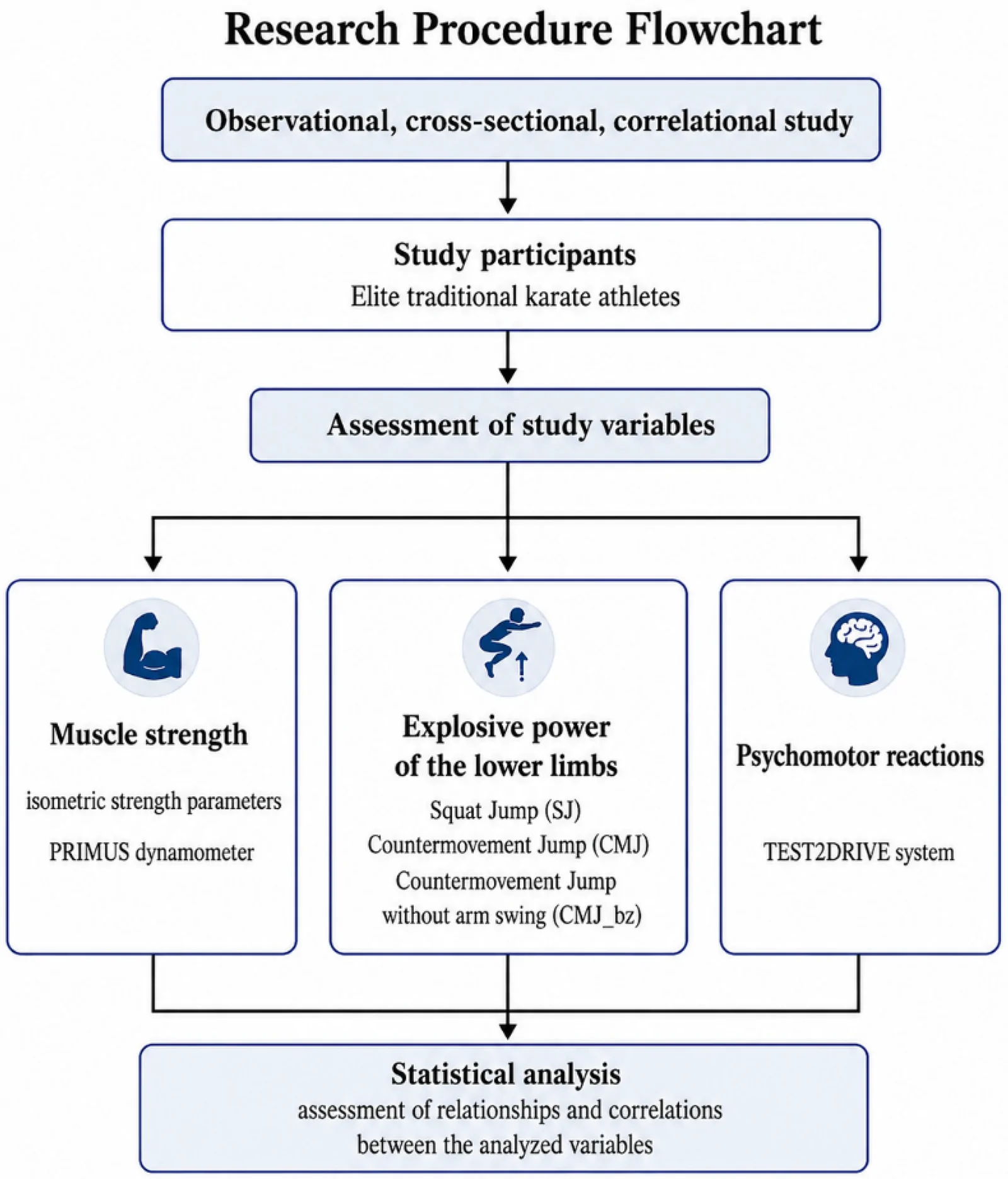
Flowchart of the research intervention.

### Participants

The study sample comprised 27 adult men who were active members of the Polish national traditional karate team and competed in national and international events. Participants were recruited using purposive sampling, and all athletes who met the inclusion criteria and were available during the recruitment period were enrolled. Given the limited size of the elite athlete population, the sample size reflected the maximum feasible recruitment from the realistically accessible population. Elite competitive status was operationally defined as current membership in the Polish national traditional karate team and active participation in national and international competitions. All participants had at least 5 years of training experience. Chronological age was calculated as the difference between the assessment date and date of birth and ranged from 18 to 30 years. Descriptive characteristics of the participants and descriptive statistics for the neuromuscular and psychomotor variables are presented in Table 1.

**Table 1:** Descriptive characteristics of the participants.

Variable	Mean	SD	Min	Max
Body Mass (kg)	72.30	9.27	54.40	95.20
Body Height (cm)	176	5.84	165	190
Body Mass Index (BMI)	23.20	2.84	18.00	30.20

The inclusion criteria were age ≥18 years, active participation in sports competition, good health status, at least 5 years of training experience, and possession of a black belt (1st dan). The exclusion criteria were current or recent musculoskeletal injury or symptoms that could affect test performance, surgery during the period preceding the study, medical contraindications to testing, use of a restrictive diet or prohibited substances, and failure to provide written informed consent. Information on chronological age, competitive status, rank, and training experience was collected using a diagnostic questionnaire administered through direct interviews with the participants and coaching staff.

### Study procedure

All measurements were conducted in the sports hall at the University of Rzeszów. Tests were performed under comparable environmental conditions and at the same time of day to minimize the influence of circadian rhythms on the results. Participants were instructed to refrain from intense physical exercise for 24 h before each testing session and to maintain their usual dietary and hydration practices. Before testing, participants were familiarized with the study procedures and the correct execution of all trials. Each athlete completed a familiarization trial for the planned tests.

Each testing session began with a standardized general and task-specific warm-up that included exercises to increase body temperature, global joint-mobility exercises, and exercises activating the muscles responsible for postural stabilization.

After the warm-up, participants completed the following sequence: (1) during the first measurement session, explosive-power tests (SJ) were performed in randomized order; (2) two days later, during the second measurement session, the psychomotor tests (TEST2DRIVE) were also completed in randomized order; and (3) during the third measurement session, isometric muscular strength was assessed using the PRIMUS device. A 48-h recovery interval separated the three measurement sessions, allowing full recovery and limiting the influence of fatigue on subsequent test results. All sessions were conducted under comparable conditions, at a similar time of day, using the same warm-up and measurement protocols. All measurements were performed by the same research team in accordance with the study protocol.

### Isometric strength assessment

The isometric strength of the upper- and lower-limb flexors and extensors was assessed bilaterally at the elbow and knee joints, respectively. The BTE PrimusRS system for objective functional assessment and training (BTE Technologies, Hanover, USA) was used. The PrimusRS is an isokinetic dynamometer that enables the assessment of isometric, isotonic, and isokinetic muscular strength (17). The axis of the dynamometer head was individually adjusted for each participant to align with the anatomical axis of rotation of the assessed joint.

The study protocol included the following measurements:

1. Isometric strength of the upper-limb flexors and extensors was assessed in a standing position. Participants stood with their feet hip-width apart, with the tested upper limb flexed to 90° at the elbow joint. The shoulders remained parallel to the floor. Participants then performed three maximal isometric contractions lasting 4 s each, first for the upper-limb flexors and then for the extensors.
2. Isometric strength of the lower-limb extensors and flexors was measured in a seated position using the specialized chair included with the BTE PrimusRS system. The tested lower limb was positioned at 90° of knee flexion, and participants kept their hands on their chest. To limit unwanted movement, the trunk, hips, and mid-thigh were stabilized with straps. Each participant performed three maximal 4-s isometric contractions, first of the lower-limb extensors and then of the flexors.

The outcome variable was isometric force expressed in newtons (N). The highest force value obtained across the three trials was used for statistical analysis.

### Lower-limb power assessment

Vertical jumps are widely used in strength and conditioning for both assessment and training. Explosive-power performance is important in many sports (18, 19). The Optojump Next system (Microgate, Bolzano, Italy) was used to measure this variable. Participants performed three types of jump: the squat jump (SJ), countermovement jump (CMJ), and countermovement jump without arm swing (CMJ_noAS). Before the actual SJ, CMJ, and CMJ_noAS measurements, participants performed 3–4 familiarization jumps to become acquainted with the testing procedure. Three repetitions of each jump were then recorded. A 1-min recovery interval was provided between repetitions, and a 2-min recovery interval was provided between jump types.

1. Squat Jump (SJ): The jump from a static position was performed from a semi-squat with approximately 90° of knee flexion. Participants lowered themselves into a squat until the knee angle reached 90° and maintained this starting position for 2–3 s to eliminate the contribution of the stretch-shortening cycle. They then performed a maximal vertical jump without a countermovement. The hands remained on the hips throughout the trial to eliminate the influence of arm movement on jump height. After take-off, participants landed on both feet while maintaining natural postural control.
2. Countermovement Jump (CMJ): The countermovement jump began from an upright standing position. Participants performed a rapid downward movement involving simultaneous flexion of the hip, knee, and ankle joints and then, without pausing, transitioned into the take-off phase and performed a maximal vertical jump. The movement was smooth and continuous, allowing use of the lower-limb stretch-shortening cycle. Participants also performed an arm swing during the jump to assist take-off and increase jump height.
3. Countermovement Jump Without Arm Swing (CMJ_noAS): The countermovement jump without upper-limb involvement was performed from a standing position. Participants made a dynamic downward movement followed by a maximal vertical take-off, while keeping their hands on their hips throughout the trial to prevent the use of an arm swing to increase jump height. The jump was performed smoothly, without pausing between lowering the center of mass and take-off, and participants landed on both feet.

Jump height was expressed in centimeters (cm), and the highest value obtained from the three valid trials for each jump type was used for statistical analysis.

### Psychomotor ability assessment

The psychomotor abilities of the karate athletes were assessed using computerized tests delivered online through the TEST2DRIVE system (ALTA, Siemianowice Śląskie, Poland) (20), TEST2DRIVE: Podręcznik użytkownika, ALTA, Katowice, Poland]. The system meets all requirements of the Regulation of the Polish Minister of Health of July 8, 2014, concerning psychological tests of psychomotor abilities. Four domain-specific tests were used to assess psychomotor performance and were completed in the following order (8, 21):

1. SIRT—simple reaction time. The stimulus signal field changed color at predetermined intervals. Participants responded by rapidly moving the finger from the Start field to the blue Reaction Time field.
2. CHORT—choice reaction time. Horizontal and vertical stimuli requiring a response, as well as neutral stimuli requiring no response, were displayed in the upper signal row. Participants moved the finger from the start field to one of two response fields corresponding to the vertical or horizontal stimulus. When a neutral stimulus was presented, no action was taken and the finger remained in the start field.
3. HECOR—hand-eye coordination. Participants moved the finger from the start field to the blue response field and then returned it to the start field.
4. SPANT—spatial anticipation. Signal fields were located at the top and on the left and right sides of the test panel. Two fields—one identifying a row and the other a column—simultaneously changed color to red. In response, participants indicated the intersection of the highlighted row and column with their finger and then returned the finger to the Start field.

After familiarization, participants completed the tasks in randomized order. They remained standing and performed the tasks using the index finger of the dominant hand. Hand dominance was determined using the pen test by observing participants as they signed the informed-consent form (8). A 20-min break was provided between tests. Testing was conducted in an isolated room to minimize distraction. During each test, stimuli appeared at varying intervals (1, 1.5, or 2 s) and were displayed for 3 s. Each test included 20 stimuli, except CHORT, which included 24 stimuli. Reaction time was measured to the nearest 0.01 s. Each test lasted 3 min and was preceded by a practice trial that enabled participants to become familiar with the procedure (6).

The psychomotor assessments used in this study were based on laboratory stimulus-response tasks and did not include karate-specific techniques, perceptual cues related to an opponent's behavior, or kinematic analysis of actual movements performed during combat. The results should therefore be interpreted as indicators of general psychomotor abilities, including components of reaction, movement initiation, and motor execution, rather than as direct measures of technical reaction performance in kumite. Consequently, any conclusions regarding the transfer of these findings to competitive performance are preliminary and require confirmation in future studies using karate-specific protocols.

### Statistical analysis

Statistical analyses were performed using PQStat version 1.8.6. Descriptive statistics were calculated as means, standard deviations, and ranges. The normality of variable distributions was assessed using the Shapiro–Wilk test. Relationships between isometric-strength variables, vertical-jump performance, and psychomotor outcomes were assessed using Pearson correlation coefficients. Because a total of 70 pairwise correlations were examined, the Benjamini–Hochberg false discovery rate (FDR) procedure was applied to control for multiple comparisons. Statistical significance after correction was defined as q < 0.05. Associations with an unadjusted *p* < 0.05 but q ≥ 0.05 were treated as nominal exploratory associations and were not interpreted as statistically significant findings. Multiple regression analyses were retained as secondary exploratory analyses. To reduce multicollinearity among the highly correlated jump-performance variables, SJ, CMJ_noAS, and CMJ were standardized using z-scores and averaged to create a single Explosive Power Composite (EPC). Multiple linear regression analyses were then performed with individual TEST2DRIVE outcomes as dependent variables and right-arm flexion and extension strength, right-leg flexion and extension strength, and EPC as predictors. R, R^2^, adjusted R^2^, F statistics, and model *p* values were calculated. Collinearity was assessed using the variance inflation factor (VIF). Given the limited sample size, the regression results were interpreted cautiously as exploratory analyses.

## Results

As shown in Table 2, most of the analyzed associations between strength variables and psychomotor reaction indicators were weak and did not reach statistical significance. At the unadjusted level, nominally significant positive correlations were found between SPANT median motor time and right-arm flexion strength (r = 0.44; *p* = 0.03), right-arm extension strength (r = 0.59; *p* = 0.002), and right-leg flexion strength (r = 0.48; *p* = 0.010). In addition, SPANT median reaction time showed negative associations at the threshold of statistical significance with right-leg flexion strength (r = −0.39; *p* = 0.050) and right-leg extension strength (r = −0.38; *p* = 0.050).

**Table 2:** Pearson correlation coefficients between muscular strength and TEST2DRIVE outcomes.

Variables	Right-arm flexion		Right-arm extension		Right-leg flexion		Right-leg extension	
	r	*p*	r	*p*	r	*p*	r	*p*
SIRT median reaction time	0.15	0.46	0.08	0.68	0.15	0.49	0.07	0.72
SIRT motor time	−0.08	0.70	−0.04	0.87	0.18	0.41	0.23	0.28
CHORT median reaction time	−0.25	0.23	−0.13	0.55	0.25	0.23	0.21	0.31
CHORT median motor time	0.05	0.80	0.24	0.25	0.33	0.12	0.18	0.39
CHORT correct responses (%)	0.06	0.77	0.09	0.66	0.09	0.67	−0.16	0.46
HECTOR median reaction time	−0.09	0.67	−0.14	0.52	−0.01	0.95	−0.05	0.79
HECTOR median motor time	0.19	0.38	0.30	0.16	0.38	0.07	0.31	0.14
SPANT median reaction time	−0.24	0.25	−0.24	0.25	**−0**.**39**	**0**.**050**	**−0**.**38**	**0**.**050**
SPANT median motor time	**0**.**44**	**0**.**03**	**0**.**59**	**0**.**002**	**0**.**48**	**0**.**010**	0.27	0.20
SPANT correct responses (%)	0.27	0.20	0.09	0.66	−0.11	0.62	0.27	0.20

r, Pearson correlation coefficient; *p*, significance level. Statistically significant correlation coefficients and their corresponding *p* values are shown in bold (*p* < 0.05).

The results presented in Table 3 indicate that the strongest and most frequently significant associations between explosive-power indicators and TEST2DRIVE outcomes involved the CHORT and HECTOR parameters. CHORT median motor time showed moderate negative correlations with SJ (r = −0.40; *p* = 0.047) and CMJ_noAS (r = −0.40; *p* = 0.049), whereas the association with CMJ reached the level of a statistical trend (r = −0.37; *p* = 0.07). The CHORT correct-response rate showed a nominal negative association with all power indicators: SJ (r = −0.51; *p* = 0.009), CMJ_noAS (r = −0.48; *p* = 0.016), and CMJ (r = −0.49; *p* = 0.013). Nominal negative correlations were also observed between HECTOR median motor time and SJ (r = −0.49; *p* = 0.01), CMJ_noAS (r = −0.45; *p* = 0.03), and CMJ (r = −0.44; *p* = 0.03). In the SPANT test, median reaction time was negatively correlated with SJ (r = −0.41; *p* = 0.047) and CMJ (r = −0.45; *p* = 0.03).

**Table 3:** Pearson correlation coefficients between explosive-power indicators (SJ, CMJ_noAS, and CMJ) and TEST2DRIVE psychomotor reaction outcomes.

Variables	SJ		CMJ_noAS		CMJ	
	r	*p*	r	*p*	r	*p*
SIRT median reaction time	0.03	0.89	0.15	0.47	−0.01	0.94
SIRT motor time	−0.35	0.09	−0.24	0.26	−0.27	0.20
CHORT median reaction time	−0.19	0.37	−0.16	0.45	−0.25	0.24
CHORT median motor time	**−0**.**40**	**0**.**047**	**−0**.**40**	**0**.**049**	−0.37	0.07
CHORT correct responses (%)	**−0**.**51**	**0**.**009**	**−0**.**48**	**0**.**016**	**−0**.**49**	**0**.**013**
HECTOR median reaction time	0.05	0.80	0.17	0.44	−0.001	0.95
HECTOR median motor time	**−0**.**49**	**0**.**01**	**−0**.**45**	**0**.**03**	**−0**.**44**	**0**.**03**
SPANT median reaction time	−0.41	0.047	−0.23	0.13	**−0**.**45**	**0**.**03**
SPANT median motor time	−0.01	0.65	−0.13	0.54	−0.03	0.89
SPANT correct responses (%)	0.15	0.49	0.23	0.27	0.19	0.38

SJ, squat jump; CMJ_noAS, countermovement jump without arm swing; CMJ, countermovement jump; r, Pearson correlation coefficient; *p*, significance level. Statistically significant correlation coefficients and their corresponding *p* values are shown in bold (*p* < 0.05).

As shown in Table 4, the multiple regression model for SPANT median motor time was statistically significant (R = 0.691, R^2^ = 0.478, adjusted R^2^ = 0.354, F = 3.844, *p* = 0.012). The models for CHORT median motor time (R^2^ = 0.366, *p* = 0.070), HECOR median motor time (R^2^ = 0.381, *p* = 0.056), and SPANT median reaction time (R^2^ = 0.356, *p* = 0.079) showed statistical trends. The remaining regression models were not statistically significant.

**Table 4:** Summary of multiple regression models for psychomotor reaction indicators obtained from the TEST2DRIVE tests.

Dependent variable (Y)	n	R	R^2^	Adjusted R^2^	F	Model p	SEE
SIRT—Median simple reaction time	27	0.315	0.100	−0.115	0.464	0.798	31.385
SIRT—Median motor time	27	0.413	0.170	−0.027	0.863	0.522	33.266
CHORT—Median choice reaction time	27	0.530	0.281	0.109	1.638	0.194	48.271
CHORT—Median motor time	27	0.605	0.366	0.215	2.420	0.070	31.320
CHORT—Correct responses (%)	27	0.537	0.289	0.119	1.705	0.177	3.370
HECOR—Median reaction time	27	0.227	0.052	−0.174	0.229	0.946	36.099
HECOR—Median motor time	27	0.618	0.381	0.234	2.590	0.056	35.055
SPANT—Median reaction time	27	0.597	0.356	0.203	2.326	0.079	60.137
SPANT—Median motor time	27	0.691	0.478	0.354	3.844	**0** **.** **012**	29.819
SPANT—Correct responses (%)	27	0.339	0.115	−0.095	0.547	0.739	10.297

n, sample size; R, multiple correlation coefficient; R^2^, coefficient of determination; adjusted R^2^, adjusted coefficient of determination; F, F statistic for the model; model p, significance level for the overall regression model; SEE, standard error of the estimate. Statistically significant values (*p* < 0.05) are shown in bold.

In the statistically significant model for SPANT median motor time, right-arm extension strength was a significant positive predictor (*β* = 0.864, *p* = 0.014). Individual coefficients observed in the remaining regression models were treated as exploratory and were not interpreted independently because the corresponding overall models did not reach statistical significance Table 5).

**Table 5:** Regression coefficients for predictors reaching statistical significance or the level of a statistical trend in the revised multiple regression models.

Dependent variable (Y)	Predictor	B	SE	Standardized *β*	t	p
CHORT—Median choice reaction time	Right-arm flexion	−1.017	0.530	−0.712	−1.921	0.068
CHORT—Median motor time	Right-arm flexion	−0.714	0.344	−0.723	−2.077	0.050
CHORT—Median motor time	Right-arm extension	0.795	0.327	0.868	2.431	**0.024**
CHORT—Median motor time	EPC	−12.088	6.740	−0.318	−1.793	0.087
CHORT—Correct responses (%)	EPC	−1.646	0.725	−0.426	−2.269	**0.034**
HECOR—Median motor time	EPC	−17.175	7.544	−0.399	−2.277	**0.033**
SPANT—Median motor time	Right-arm extension	0.831	0.311	0.864	2.669	**0.014**

B, unstandardized regression coefficient; SE, standard error; standardized β, standardized regression coefficient; t, t statistic; p, statistical significance level; EPC, Explosive Power Composite. Statistically significant values (*p* < 0.05) are shown in bold. Values of 0.05 ≤ *p* < 0.10 were interpreted as statistical trends.

Following the replacement of the three individual jump variables with the Explosive Power Composite (EPC), no problematic multicollinearity was observed. VIF values ranged from 1.04 to 4.22, with the lowest value observed for EPC (VIF = 1.04). These results indicate that the revised predictor set provided an acceptable level of independence among predictors (Table 6).

**Table 6:** Variance inflation factor (VIF) values for predictors included in the revised multiple regression models.

Predictor	VIF
Right-arm flexion	4.01
Right-arm extension	4.22
Right-leg flexion	1.47
Right-leg extension	1.48
EPC	1.04

EPC, Explosive Power Composite calculated as the mean of standardized z-scores for SJ, CMJ_noAS, and CMJ; VIF, variance inflation factor.

## Discussion

The present exploratory study identified several moderate associations between selected neuromuscular and psychomotor variables at the unadjusted level. However, none of the 70 correlations remained statistically significant after correction for multiple comparisons. Accordingly, the findings should not be interpreted as confirmatory evidence that either isometric strength or vertical-jump performance is independently associated with psychomotor performance in elite kumite athletes. Rather, the observed patterns should be considered preliminary and hypothesis-generating and require confirmation in larger samples using more sport-specific measures (22, 23).

The specific demands of the discipline must be considered when interpreting these findings. Kumite is an open-skill event characterized by high decision-making demands, in which performance depends not only on strength or power potential but also on anticipation, rapid recognition of the situation, response selection, and the quality of movement execution under time pressure. The literature emphasizes that karate athletes, particularly at the elite level, display a profile characterized by well-developed power, speed, and perceptual-decision-making competence. In kumite, the ability to execute actions rapidly and explosively is considered more important than the isolated capacity to generate high maximal force (22, 23). Recent evidence from members of the traditional karate national team has also reported selective relationships between somatic and psychomotor characteristics, further illustrating the complexity of these associations in highly trained athletes (24).

The method used to assess strength in the present study is particularly relevant to the interpretation of the findings. Strength parameters were obtained under isometric contraction conditions, that is, in a static measurement setting, at a defined joint position, and without joint movement. Such measurements provide valuable information about maximal force-generating capacity under controlled conditions but are not equivalent to dynamic force production during ballistic movement. Available evidence indicates that isometric parameters may be related to dynamic-task performance; however, the magnitude of these relationships depends on the specific test, the variable analyzed, and the similarity between the measurement conditions and the target motor task (25, 26). Isometric and dynamic strength measures should therefore not be considered interchangeable (27). In the present study, the largely nonsignificant relationships between isometric strength and psychomotor outcomes suggest that these laboratory assessments captured distinct aspects of performance rather than directly corresponding capacities.

At the unadjusted level, greater isometric strength in selected measures was associated with longer motor time in the SPANT task. Thus, stronger athletes did not necessarily demonstrate faster movement execution in this laboratory-based psychomotor task. This direction of association should be addressed directly rather than interpreted as evidence of improved psychomotor performance. One possible explanation is the low task specificity between maximal isometric force production and a computerized task requiring rapid movement of the index finger; however, the present study was not designed to identify the mechanism underlying this relationship. Importantly, these associations did not remain statistically significant after correction for multiple comparisons. Consequently, the present findings do not provide sufficient evidence for a direct relationship between maximal isometric strength and psychomotor response speed (26).

At the unadjusted level, selected vertical-jump measures showed negative associations with motor or reaction times in some TEST2DRIVE outcomes. These observations indicate that higher jump performance coincided with shorter response times in selected laboratory tasks. However, these associations did not remain statistically significant after correction for multiple comparisons and therefore should not be interpreted as evidence that greater lower-limb explosive performance improves psychomotor performance. Furthermore, vertical-jump tests primarily assess lower-limb function, whereas the TEST2DRIVE tasks used in the present study require rapid movements of the index finger. Accordingly, the observed cross-domain associations may reflect broader characteristics of the athletes rather than a direct biomechanical or functional transfer between lower-limb performance and finger-response speed. Previous studies have demonstrated the importance of explosive capabilities for karate performance (23, 28), but the present results do not establish that such capabilities directly determine performance in computerized psychomotor tasks (23, 28).

The negative correlations between explosive-power indicators and the percentage of correct responses in the CHORT test warrant separate consideration. Descriptively, this pattern might suggest that more powerful athletes completed the task faster but at the expense of accuracy. This pattern may be interpreted in the context of the speed–accuracy trade-off, whereby a strategy favoring rapid response initiation and execution may increase the probability of response errors. In highly trained combat-sport athletes, repeated exposure to situations requiring immediate action may promote a response strategy that prioritizes speed under time pressure. However, the present cross-sectional data do not allow us to determine whether greater explosive power itself contributes to such a strategy or whether both characteristics reflect broader adaptations associated with elite kumite training. This interpretation should not, however, be stated categorically. First, the relationship was limited to one type of task and was not consistently replicated across the full test profile. Second, computerized tasks have limited ecological validity relative to actual combat situations, in which stimuli are more information-rich and embedded in the context of an opponent's movements. Third, previous studies have shown that the advantage of karate experts is especially evident in tasks involving response selection and anticipation rather than merely rapid reactions to abstract stimuli (20, 29). It is therefore more appropriate to conclude that the negative association with response accuracy may reflect a task-specific laboratory performance strategy rather than a genuine athletic “cost” of greater explosive power (20).

It is also noteworthy that the fewest associations were observed for the SIRT simple reaction task. This finding is not unexpected in light of the literature. Mori et al. demonstrated that the advantage of karate athletes over untrained individuals is substantially more evident in choice-reaction and anticipation tasks than in simple reaction tasks (20). The meta-analysis by Zhang et al. further confirmed that differences between experts and novices in combat sports become more apparent when the stimuli resemble real sport situations (29). Simple reaction time may therefore be insufficiently sensitive to detect functionally relevant differences in elite kumite, particularly in a sample composed of athletes with a very high competitive level. In such a group, tasks engaging selection, anticipation, and execution-control processes are likely to have greater diagnostic value (20, 29).

The secondary exploratory regression analysis identified one statistically significant overall model for SPANT median motor time. In this model, right-arm extension strength was a significant positive predictor. The remaining regression models did not reach statistical significance. Replacement of the three highly correlated jump variables with the Explosive Power Composite substantially reduced multicollinearity, with VIF values ranging from 1.04 to 4.22. Nevertheless, given the limited sample size, the regression findings should be interpreted cautiously as exploratory and require confirmation in larger samples (30, 31).

The present findings are consistent with a broader understanding of physical preparation in karate athletes. Isometric strength is not irrelevant; on the contrary, it is an important component of neuromuscular potential and may support stabilization, force-impulse generation, and energy transfer through the kinetic chain. However, for psychomotor tasks more directly related to movement speed, capacities reflecting rapid force development and the ability to execute movement at high velocity may be more important. The review by Lum et al. indicates that isometric parameters are informative and diagnostically useful, but their interpretation should consider whether peak force, impulse, or rate of force development is being analyzed (26). Maffiuletti et al. similarly emphasized that, in explosive tasks, rate of force development—the ability to activate the neuromuscular system rapidly during the first tens of milliseconds of contraction—may be functionally more relevant than maximal strength alone (15).

From a practical perspective, psychomotor performance in elite kumite should not be predicted solely on the basis of maximal isometric strength. Such measurement may be a valuable component of strength-preparation monitoring but should not be treated as an independent predictor of response quality in choice-reaction tasks. A more appropriate approach is to combine the assessment and development of explosive power with perceptual-decision-making tasks, reactions to stimuli with high sport-specific validity, and training aimed at increasing the execution speed of specific techniques. Among elite athletes with similar levels of preparation, the integration of motor and cognitive-perceptual characteristics may determine subtle functional differences (28, 29).

In the studied members of the Polish national traditional karate kumite team, maximal isometric strength assessed using the PRIMUS dynamometer showed limited and selective associations with psychomotor-test outcomes, whereas explosive power was associated with these outcomes more frequently and consistently. The most plausible interpretation is that functional advantage in elite kumite depends less on the isolated ability to generate high force under static conditions than on the ability to express that force rapidly during dynamic, complex, and decisionally demanding actions (15, 26, 28). At the same time, the absence of significant multiple regression models warrants caution and suggests that psychomotor performance at this competitive level depends on the interaction of multiple factors rather than on a single motor characteristic considered independently of the task context (30, 31).

### Study limitations

The present study has several limitations. Its cross-sectional design precludes causal inference, and the relatively small sample of 27 male national-team athletes limits statistical power and generalizability. In addition, multiple correlations were examined, and none remained statistically significant after FDR correction, so the findings should be considered exploratory. The neuromuscular and psychomotor tests also involved different motor effectors and were not specific to kumite performance. Finally, isometric strength was assessed as absolute force without normalization to body mass, and potential effects of handedness were not analyzed separately. Future studies should include larger and more diverse samples, body-mass-normalized strength measures, and more sport-specific reaction tasks.

## Conclusions

1. In elite traditional karate kumite athletes, significant relationships were identified between selected neuromuscular parameters and psychomotor performance; however, these relationships were selective and depended on the specific outcome analyzed.
2. Explosive-power indicators showed more frequent and consistent associations with psychomotor reaction parameters than isometric strength, suggesting greater functional relevance of rapid force production to selected components of motor-response performance.
3. After combining SJ, CMJ_noAS, and CMJ into a single Explosive Power Composite (EPC) to address multicollinearity, the regression model for SPANT median motor time was statistically significant, while several additional models showed statistical trends.
4. The revised regression analyses indicated that selected neuromuscular variables, including explosive power and upper-limb isometric strength, may contribute to specific psychomotor outcomes, although these relationships were not consistent across all TEST2DRIVE measures.
5. Psychomotor performance in elite kumite athletes appears to reflect the interaction of multiple neuromuscular and coordinative factors rather than dependence on a single isolated physical characteristic.

### Practical applications

1. In the training process of elite kumite athletes, explosive-power development should be considered alongside perceptual and decision-making training, particularly in tasks requiring rapid response initiation and movement execution under time pressure.
2. Isometric-strength assessment may be used as one component of neuromuscular profiling; however, it should not be interpreted as an independent indicator of psychomotor performance.
3. Athlete monitoring and training design should integrate physical and psychomotor assessments, including measures of explosive power, reaction time, motor time, and response accuracy.
4. Training programs may benefit from incorporating exercises that combine rapid force production with sport-specific perceptual and decision-making demands, while recognizing that the present findings demonstrate associations rather than causal effects.

## Data Availability

The original contributions presented in the study are included in the article/Supplementary Material, further inquiries can be directed to the corresponding author.
